# Surgical Management of Traumatic Brain Injury Based on Intracranial Compliance: Toward Personalized Decision-Making

**DOI:** 10.3390/brainsci16050538

**Published:** 2026-05-20

**Authors:** Santiago Cardona-Collazos, Laura M. Loaiza-Cardona, Andres Salazar-Restrepo, Luigi Valentino Berra, Andres M. Rubiano

**Affiliations:** 1 Meditech Foundation, Cali 760031, Colombia; cardona.santiago.meditechf@outlook.com (S.C.-C.); loaiza.laura.meditechf@outlook.com (L.M.L.-C.); ansalazar1207@gmail.com (A.S.-R.); 2 Department of Neurosurgery, Policlinico Umberto I, Sapienza University, 00161 Rome, Italy; luigivalentinoberra@gmail.com; 3 Neuroscience Institute, El Bosque University, Bogota 110121, Colombia; 4 Department of Neurosurgery, Universidad del Valle, Cali 76001, Colombia

**Keywords:** traumatic brain injury, neurosurgery, neurotrauma, neurosurgical procedures, intracranial compartment syndrome, intracranial compliance, precision medicine

## Abstract

**Highlights:**

**What are the main findings?**

• The framework of surgical management of the intracranial compartment syndrome shifts surgical reasoning from lesion-centered thresholds to physiology-centered management.
• What ultimately drives the neurosurgical intervention are not numerical values, but the loss of intracranial compliance and the physiological consequences that follow.

**What are the implications of the main findings?**

• This framework proposes a surgical shift from traditional approaches to new models based on physiopathology instead of primary injury size.
• This compliance-based surgical model does not challenge randomized evidence but offers a physiological lens through which existing surgical outcome data can be interpreted more coherently, helping explain outcome variability, refining patient selection, and guiding future trial design.

**Abstract:**

Traditional surgical decision-making in traumatic brain injury (TBI) has relied on static intracranial pressure (ICP) thresholds and fixed volumetric criteria, an approach that inadequately reflects the dynamic physiological nature of secondary brain injury. These conventional metrics fail to capture the critical determinant of clinical deterioration: the progressive loss of intracranial compliance, the brain’s capacity to buffer additional volume without harmful pressure escalation. This manuscript proposes a practical, compliance-based framework for selecting precise, personalized surgical strategies using real-time physiological, imaging, and neuromonitoring indicators. Based on the Intracranial Compartment Syndrome (ICCS) model, this approach translates the loss of compensatory reserve into actionable operative decisions. Compliance is assessed through multimodal tools, including ICP waveform morphology, cerebral oxygenation, and complementary noninvasive neuromonitoring. ICCS staging delineates three operative contexts: Stage 1, preserved compliance; Stage 2, compliance failure with maintained oxygenation requiring physiology-guided interventions to restore buffering capacity; and Stage 3, global decompensation with lost of compliance plus oxygenation failure requiring immediate, aggressive intervention for partial or total brain tissue survival. By shifting surgical reasoning from fixed anatomical thresholds to a physiology-centered assessment of intracranial compliance, this framework aims to enhance the timing, selection, and overall effectiveness of neurosurgical interventions in TBI.

## 1. Introduction: Why Neurosurgeons Need a New Decision Framework

Traumatic brain injury (TBI) remains one of the leading global causes of morbidity and mortality, affecting more than 60 million individuals annually and disproportionately impacting young, economically active populations [1,2]. TBI accounts for approximately one-third of all injury-related deaths worldwide, with an estimated annual incidence ranging from 47 to 849 per 100,000 population across different regions. High-income countries report road traffic collisions, falls, and sports injuries as predominant mechanisms, whereas low- and middle-income countries bear a disproportionate burden due to limited neurotrauma infrastructure and delayed access to specialized care [1,2]. Despite advances in critical care and surgical techniques, case fatality rates for severe TBI remain high, and a substantial proportion of survivors experience permanent cognitive, behavioral, and neurological deficits that represent an enormous personal and socioeconomic burden. The lethality is driven not only by the mechanical forces that produce the primary injury but, more importantly, by the cascade of cerebral edema, ischemia, and physiological deterioration that constitutes secondary brain injury [3,4]. For decades, ICP management and consequently, surgical decision-making, have been based on the classical Monro–Kellie doctrine and on static thresholds of ICP or primary injury volume. This traditional framework shaped widely adopted guidelines and algorithms that continue to influence global neurotrauma practice [5,6,7,8].

However, contemporary research has revealed that intracranial dynamics is far more complex than what can be captured by fixed ICP cut-offs or volumetric criteria alone. Updated models of intracranial physiology highlight the importance of cerebral autoregulation, venous outflow, cerebrospinal fluid (CSF) circulation, and the glymphatic system, all of which directly influence the brain’s ability to tolerate volume changes [9,10,11,12,13,14]. Within this expanded understanding, intracranial compliance, defined as the capacity of intracranial compartments to tolerate increases in volume without generating harmful rises in pressure, emerges as a central determinant of physiological vulnerability.

The concept of the ICCS integrates these modern principles into a clinically actionable framework. ICCS identifies the point at which compensatory mechanisms are exhausted and intracranial compliance is lost, signaling a transition toward impaired cerebral perfusion, reduced brain oxygenation, and imminent risk of secondary injury [15,16,17,18]. Importantly, ICCS allows clinicians to characterize the real-time compliance of each patient’s intracranial cavity, acknowledging that individuals with similar ICP values or lesion sizes may exhibit profoundly different physiological trajectories.

This shift has profound implications for surgery. Traditionally, recommendations for craniotomy, external ventricular drainage, expansion craniotomy or decompressive craniectomy have been informed by fixed lesion dimensions or ICP thresholds. Current evidence increasingly suggests that these criteria do not fully reflect the underlying pathophysiology [19,20,21,22,23,24,25,26,27]. Instead, surgical interventions should be selected according to the patient’s stage of intracranial compliance failure, integrating imaging findings, multimodal neuromonitoring, and physiological indicators. Under this perspective, surgery becomes a dynamic, staged process tailored to the specific profile of intracranial compartment dysfunction, before the brain tissue suffers irreversible damage.

This article proposes a practical, compliance-based model for surgical decision-making in TBI. Drawing on the ICCS framework, it outlines how neurosurgeons can integrate pathophysiology, imaging, and neuromonitoring to select precise and personalized interventions tailored to the patient’s intracranial compliance. By reframing surgical strategies through the lens of compliance rather than static thresholds, this approach aims to align operative treatment more closely with individual physiological needs and to enhance the precision and effectiveness of neurotrauma surgery.

## 2. Surgical Problem: What Surgeons Are Actually Targeting in TBI

In TBI, the true surgical target is not simply the size of the hematoma or even the elevated ICP value. Neurosurgeons are fundamentally treating the loss of intracranial compliance, the point at which an intracranial compartment can no longer absorb additional volume without producing harmful pressure rises or compromising cerebral perfusion. This physiological collapse is defined by the ICCS concept.

**Figure 1:**
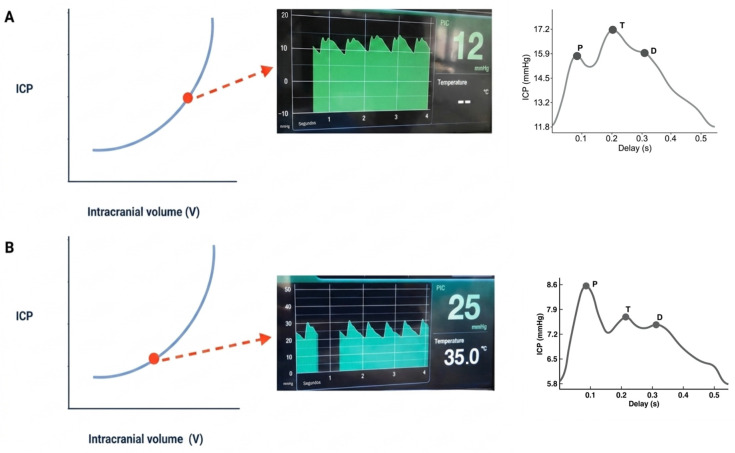
Two monitors displaying invasive neuromonitoring results using intraparenchymal catheters in patients with TBI. Panel (**A**) shows an ICP waveform with impaired intracranial compliance, despite an ICP value below the standard intervention threshold of 22 mmHg. The waveform morphology, characterized by a P2 > P1, suggests a decreased compensatory reserve. In contrast, Panel (**B**) shows a waveform consistent with preserved compliance, displaying a well-defined triphasic morphology even in the presence of an ICP value over the normal threshold. Both waveforms are correlated with their respective positions along the intracranial volume–pressure curve, showing in both inconsistencies with the current framework for ICP-driven management. The letters P, T, and D denote the percussion wave (P1), tidal wave (P2), and dicrotic wave (P3) of the ICP waveform, respectively. Adapted from [16].

TBI initiates a complex secondary injury cascade, including various types of cerebral edema, venous outflow obstruction, impairment of CSF circulation and glymphatic clearance, and progressive dysregulation of cerebrovascular autoregulation, that incrementally consumes the intracranial compensatory reserve. As compliance deteriorates, small changes in intracranial volume induce larger pressure shifts, marking a state of critical vulnerability [10]. Importantly, equivalent ICP measurements or comparable lesion volumes may reflect distinct compliance states across patients, highlighting the substantial interindividual variability in intracranial compliance and the inherent limitations of relying on static pressure or volumetric thresholds for surgical decision-making [10,25] (Figure 1).

From a surgical perspective, this means that operative interventions look for reversing an intracranial compliance failure, and not only to remove a mass effect. Hematoma evacuation, CSF diversion, cisternal opening, and decompressive craniectomy all work by restoring buffering capacity and protecting cerebral perfusion [10,21,22,25,28,29,30,31].

Thus, the surgical problem in TBI is ultimately recognizing and intervening upon the evolving stages of intracranial compliance exhaustion. Within this framework, surgical interventions become sequential, physiology-guided steps within a therapeutic hierarchy, selected according to the patient’s real-time compliance stage rather than predefined volumetric or pressure cutoffs. Ultimately, each operative option is chosen for its ability to restore, redistribute, or create additional intracranial volume reserve for increase compliance. This issue aligns surgical decision-making with the pathophysiology of ICCS and enables a more precise, patient-specific approach.

## 3. Intracranial Compartment Syndrome for Surgeons

**Figure 2:**
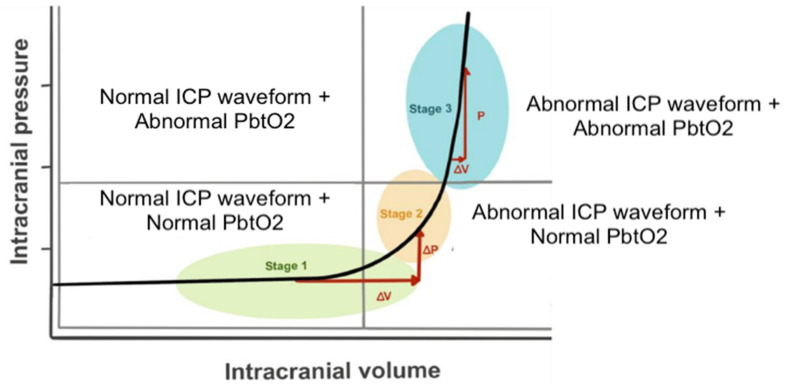
The progressive stages of ICCS show different points along the intracranial volume–pressure curve. Each stage reflects changes on intracranial compliance, with downstream effects on cerebral perfusion and cerebral oxygenation. As compliance decreases, small increases in intracranial volume result in disproportionately higher intracranial pressure, compromising cerebral hemodynamics and oxygen delivery. Adapted from [16].

For surgeons, the ICCS framework functions as a practical tool for translating intracranial physiology into operative strategy. Rather than redefining the mechanisms of compliance failure, ICCS provides a clinically actionable representation of it: a progressive loss of the ability to accommodate small increases in intracranial volume, whereby increasingly minor lesions, shifts, or edema loads produce disproportionately greater physiological consequences [15,16,17,32] (Figure 2).

By staging this progression, understanding the ICCS principles allows the surgeon to differentiate when the problem can be controlled with less aggressive procedures (e.g., hematoma evacuation, CSF diversion) and when any of the compartments require full decompression. This staging remains useful even without invasive ICP monitoring, as it integrates multimodal surrogates of compliance, such as non-invasive neuromonitoring tools (pupillometry, transcranial Doppler, non-invasive ICP waveform analyzer, optic nerve sheath diameter, and brain oxygenation) to approximate the real state of intracranial compliance. As additional monitoring modalities are incorporated, the surgeon gains a more accurate approximation of the patient’s true compliance state, enabling finer surgical discrimination and more precisely targeted intervention.

Ultimately, the ICCS concept offers a decision-making scaffold: a way to scale operative interventions to the degree of compartment compromise, ensuring that surgical choices respond to the evolving loss of intracranial compliance rather than to static radiological or pressure metrics. By providing an earlier and more physiologically grounded objective signal of decompensation, ICCS principles enable the surgeon to justify and anticipate surgical intervention before overt clinical deterioration occurs, a point at which cellular and structural injury is often already underway and may no longer be fully reversible.

## 4. Compliance Assessment and Intracranial Compartment Syndrome Staging

Intracranial compliance assessment determines the patient’s position on the pressure–volume curve. The most direct method is ICP waveform analysis via invasive monitoring. A normal waveform with P1 > P2 > P3 indicates preserved compliance, whereas P2 predominance reflects reduced buffering capacity [33,34].

Cerebral oxygenation monitoring (invasive or non-invasive) contributes to physiological stratification. When invasive ICP monitoring is unavailable, compliance is estimated using non-invasive surrogates, including pupillometry, optic nerve sheath diameter (ONSD), non-invasive ICP waveform analyzer, transcranial Doppler indices (PI, FVd), and tissue oxygenation [35].

ICCS staging reflects progressive intracranial decompensation. Stage 1 denotes structural injury with preserved compliance. Stage 2 identifies established compliance failure with preserved cerebral oxygenation and represents the optimal window for timely surgical intervention. Stage 3 indicates global decompensation with impaired oxygen delivery and requires urgent decompression. Table 1 shows the relevant findings for each ICCS stage [32].

ICCS stages function as a surgical stratification system, determining not only whether intervention is required, but also the aggressiveness of the surgical intervention that is needed.

## 5. Surgical Principles for Intracranial Compartment Syndrome Stage

### 5.1. Stage 1: Structural Injury with Preserved Compliance

**Principle**: When intracranial compliance is preserved, surgery aimed at compliance restoration or cranial decompression is not indicated.

**Surgical intent**: At this stage, surgery is limited to:

• **Infection control** (e.g., penetrating injuries, post-traumatic cerebrospinal fluid leaks);
• **Anatomical restoration** (e.g., depressed skull fractures);
• **Neuromonitoring procedures** (e.g., intraparenchymal monitoring, EVD).

**Rule**: Compliance-oriented or decompressive surgery should not be performed unless documented compliance loss and/or clinical deterioration is present, as such intervention increases morbidity without producing meaningful physiological benefit.

### 5.2. Stage 2: Compliance Failure with Preserved Oxygenation

**Principle**: Once intracranial compliance is compromised, surgical options should be actively considered and individualized according to the patient’s physiological profile, independent of classical ICP cutoffs or imaging-based volumetric thresholds. Operative strategies should be guided by physiology rather than static numerical values; depending on the clinical context, a brief period of optimized medical management may be appropriate before definitive surgical escalation.

**Surgical intent**: Restore intracranial buffering capacity to halt the progression toward perfusion failure, ischemia, and hypoxia.

**Rules**:

• Surgical indication is physiology-driven rather than volume-based or dictated by fixed ICP cutoffs;
• Space-occupying lesions may require evacuation below classical thresholds if they affect the intracranial compliance;
• Surgical treatment of intracranial hypertension may be required when medical management fails, and compliance remains compromised;
• Procedures are selected according to the dominant mechanism of compliance failure: focal mass effect, regional loss of buffering, or impaired CSF circulation. It can start with a simple ventriculostomy or a cisternostomy approach to a craniotomy procedure;
• At this stage, delay converts a correctable physiology into irreversible injury.

**Escalation triggers** (if any are present, the operative mindset must transition to Stage 3 strategy)

• Midline shift ≥5 mm;
• Peri mesencephalic cistern effacement (Grade III edema);
• Acute subdural hematoma with Zumkeller Index > 3;
• Compromised cerebral autoregulation;
• Penetrating brain injury.

### 5.3. Stage 3: Compliance Failure with Impaired Oxygenation

**Principle**: Once intracranial compliance and cerebral oxygenation are compromised, the physiological threshold for definitive decompressive intervention is reached, and conservative measures alone are insufficient to reverse ischemia. The brain is already ischemic, and any delay in definitive intervention converts salvageable tissue into established infarction. Partial measures that fail only consume critical time. Therefore, the operative strategy must be decisive and aggressive, with a low threshold for cranial decompression. Landmark randomized trials (DECRA, RESCUEicp) demonstrated complex trade-offs between mortality and functional outcomes following decompressive craniectomy [20,21], underscoring that aggressive decompression does not uniformly produce favorable neurological recovery. Within the ICCS framework, Stage 3 interventions are reserved for patients with documented compliance failure combined with oxygenation compromise, where the physiological rationale is most compelling. This conceptual staging is intended to guide clinical reasoning, not to replace individualized judgment or override the nuanced interpretation of existing trial data. In the setting of ischemia and hypoxia, delayed cerebral swelling should be anticipated. Therefore, transient intraoperative brain relaxation does not indicate stable compliance, and limited decompression risks early postoperative failure. At this stage, surgery is not preventive; it is tissue salvation.

**Surgical intent**: To rescue ischemic but viable brain from infarction through rapid restoration of cerebral perfusion.

**Rules**:

• Surgical intervention is mandatory once compliance failure is accompanied by impaired cerebral oxygenation not attributable to extracranial causes.
• Definitive and aggressive decompression is favored over temporizing or partial procedures that risk transient or incomplete restoration of cerebral perfusion. The threshold for cranial decompression should remain low.
• Apparent intraoperative brain relaxation must not be interpreted as restored compliance or as justification to avoid decompression. Surgical planning must anticipate delayed edema secondary to ischemia–hypoxia injury.
• At this stage, surgery is not preventive; it looks for partial or total brain tissue recovery. Delay converts an ischemic brain into an infarcted brain.
• Procedures include expansion craniotomy (hinge craniotomy) or full decompression.

## 6. A Practical Surgical Decision-Making Framework

The ICCS-based model replaces lesion-driven thinking with a compliance-driven surgical doctrine. Instead of asking “How large is the hematoma?” or “What is the ICP?”, the operative question becomes: Can the intracranial compartment tolerate the current volume, and any additional volume, without compromising perfusion? And this can be assessed through intracranial compliance evaluation.

This framework places intracranial compliance at the center of surgical reasoning, enabling a personalized, physiology-based approach to each patient’s injury rather than reliance on fixed anatomical thresholds.

**Figure 3:**
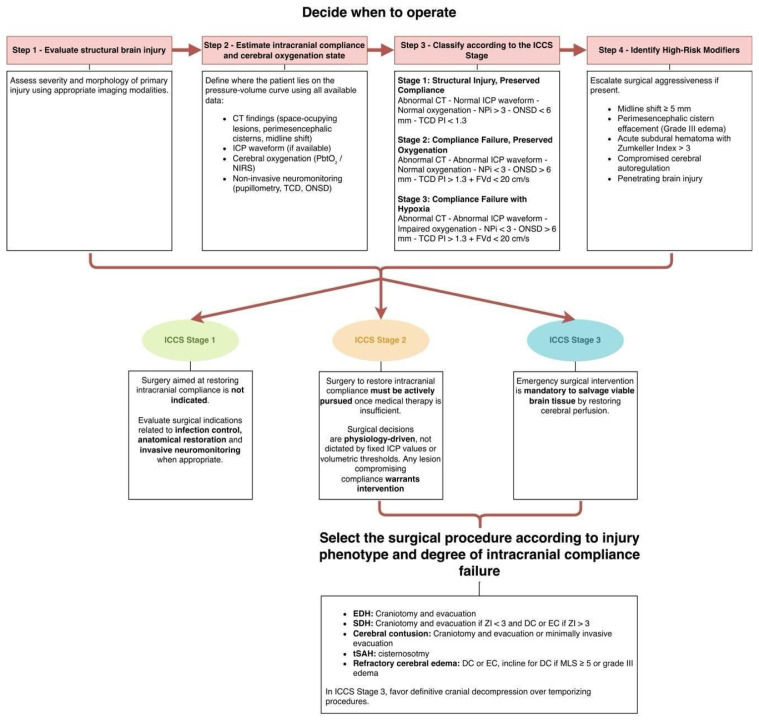
**Compliance-Based Surgical Workflow for Intracranial Compartment Syndrome in TBI**. This figure illustrates a compliance-driven surgical decision process for traumatic brain injury based on the ICCS framework. Management is organized according to physiological stages defined by intracranial buffering capacity and cerebral oxygenation rather than absolute ICP thresholds or lesion volume. Structural injury is first assessed through neuroimaging, followed by estimation of intracranial compliance using ICP waveform morphology, cerebral oxygenation, and non-invasive neuromonitoring surrogates. Patients are classified into ICCS stages, which determine surgical urgency and aggressiveness: Stage 1 favors structural or preventive interventions; Stage 2 prioritizes compliance restoration before perfusion failure occurs; and Stage 3 mandates emergent decompression to recover brain tissue. High-risk modifiers override baseline stage and prompt escalation. The model emphasizes physiology-guided surgical strategy over static criteria. EDH = Epidural Hematoma; SDH = Subdural hematoma; tSAH = Traumatic Subarachnoid Hemorrhage; EC = Expansive Craniotomy; DC = Decompressive Craniectomy; MLS = Midline Shift; PbtO_2_ = Partial Brain Tissue Oxygen Pressure.

Figure 3 illustrates the ICCS-based, compliance-driven surgical workflow for decision-making in traumatic brain injury proposed in this manuscript. This framework does not contradict existing surgical evidence but rather provides a physiological structure to interpret it.

## 7. Selected Scenarios (Pearls and Pitfalls)

### 7.1. Decompressive Craniectomy

**Goal**:

To remove the rigid cranial constraint from the pressure–volume relationship, allowing substantial intracranial volume expansion to restore and preserve cerebral perfusion within the decompressed compartment.

**Situations where it works best**:

• ICCS Stage 3 is driven by any combination of primary injuries, particularly refractory cerebral edema despite optimized medical and prior surgical management;
• Prolonged hypoxic–ischemic injury of any origin, even in the absence of marked swelling at the time of intervention, anticipating delayed cerebral edema;
• Presence of high-risk modifiers (e.g., severe midline shift, cisternal obliteration, Zumkeller index > 3).

**Figure 4:**
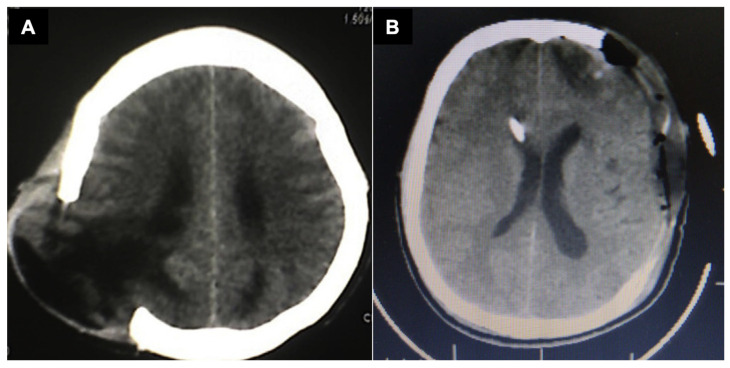
Panel (**A**) shows a CT scan demonstrating inadequate decompression with associated brain herniation (“fungus cerebri”) and established cerebral infarction due to insufficient cranial expansion. Limited craniectomies fail to achieve meaningful intracranial volume augmentation and are associated with a higher risk of secondary injury and poor neurological recovery in severe traumatic brain injury. Common complications include cortical infarction, venous congestion or bleeding, external herniation (“fungus cerebri”), and subdural hygromas. Panel (**B**) shows a CT scan demonstrating a well-performed decompressive craniectomy. There is a large frontal-temporal-parietal bone flap removal (anteroposterior diameter ≥12 cm) with adequate basal temporal decompression.

**Pitfalls**:

• Insufficient bone removal leading to incomplete decompression, particularly along the middle cranial fossa floor (see Figure 4);
• Misinterpreting transient intraoperative brain relaxation as evidence of sustained compliance restoration;
• Delayed decompression after irreversible cerebral hypoperfusion and infarction have already occurred;
• Inadequate or overly tight duroplasty that limits postoperative expansion.

**Surgical pearls:**

**Figure 5:**
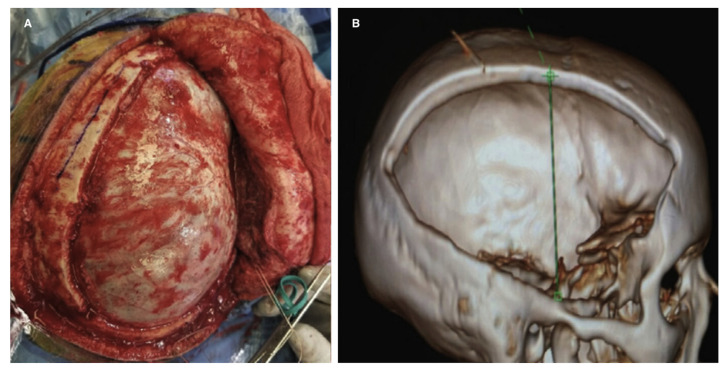
Panel (**A**) shows an intraoperative view of a wide frontal-temporal-parietal decompressive craniectomy before dural opening. Panel (**B**) displays a three-dimensional CT reconstruction demonstrating effective decompression of the anterior and middle cranial fossae, with adequate release of the temporal floor and frontal base to achieve meaningful intracranial volume expansion.

**Figure 6:**
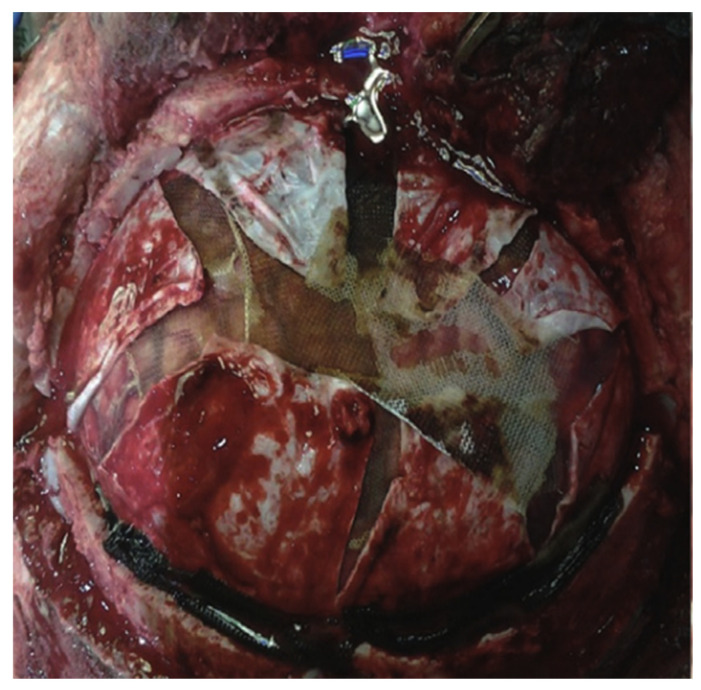
Intraoperative view of a wide frontal-temporal-parietal decompressive craniectomy demonstrating stellate dural opening, which permits uniform cortical expansion and enhances compliance gain by eliminating residual dural constraint.

**Figure 7:**
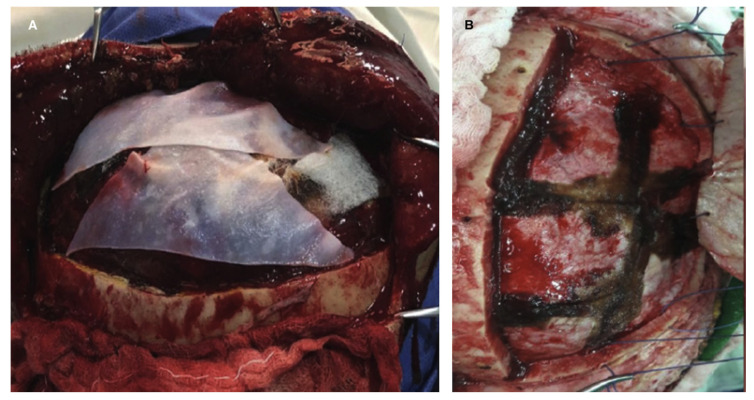
Alternative dural coverage strategies when watertight duroplasty is not feasible. Panel (**A**) demonstrates the use of a dural substitute, while Panel (**B**) shows the application of a hemostatic overlay to protect the cortical surface without restricting postoperative expansion.

• Favor wide frontal-temporal-parietal craniectomies (≥12–15 cm) over limited exposures (see Figure 5);
• Ensure adequate decompression of the frontal base and temporal floor to maximize volumetric gain;
• Choose dural opening patterns that maximize surface exposure and relaxation. C-shaped and stellate durotomies provide superior decompression by allowing uniform cortical expansion and minimizing residual dural constraint compared to limited linear or H-shaped incisions (see Figure 6);
• Match dural expansion to the extent of bone removal; avoid constrictive duroplasty;
• Watertight dural closure will not always be possible. Dural substitutes or hemostatic wraps may be used to provide cortical protection without restricting postoperative expansion (see Figure 7);
• Anticipate postoperative edema: minimal brain bulge intraoperatively does not predict postoperative compliance.

### 7.2. Expansion Craniotomy

**Goal**:

To augment the volume of a supratentorial compartment to improve and/or restore and preserve cerebral perfusion within the decompressed compartment.

**Situations where it works best**:

• ICCS Stage 2 driven entirely or in part by mild to moderate refractory cerebral edema despite optimized medical and prior surgical management.

**Pitfalls**:

• Underestimating the required volumetric expansion, resulting in insufficient compliance gain;
• Fixating the bone flap too close to its original plane, eliminating the intended space for cerebral expansion;
• Mistaking transient intraoperative brain relaxation for long-term physiological improvement when prolonged ischemia has occurred;
• Delaying conversion to decompressive craniectomy when compliance continues to deteriorate.

**Figure 8:**
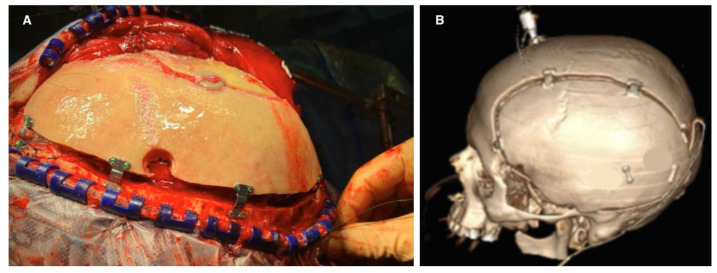
Panel (**A**) shows an intraoperative view of an expansion craniotomy with specialized fixation plates in place, demonstrating elevation of the bone flap above the cranial rim. Panel (**B**) presents a postoperative three-dimensional CT reconstruction confirming the maintained elevation of the bone flap and the appropriate positioning of the fixation hardware.

**Surgical pearls**:

• In cases of expansion craniotomy or hinge craniotomy, elevate the bone flap at least 10–15 mm above the external table cranial edge to achieve meaningful volume expansion;
• Secure rigid fixation (plates or hinges) that maintains stable outward displacement (see Figure 8);
• Avoid watertight or constrictive duroplasty; dural slack is essential for effectiveness;
• Re-evaluate intraoperatively: if expansion is insufficient, escalate promptly to decompressive craniectomy.

### 7.3. Cisternostomy

**Goal**:

To restore CSF dynamics by decompressing the basal cisterns and re-establishing pressure gradients between the subarachnoid space and the interstitial compartment, thereby reversing CSF-shift edema and improving intracranial compliance.

**Situations where it works best**:

• ICCS Stage 2 driven by basal cisternal crowding by tSAH with suspected obstruction of CSF flow.

**Pitfalls**:

• Using cisternostomy in the setting of established ischemia and hypoxia as a substitute for decompressive craniectomy. Cisternostomy should be considered a compliance-modifying strategy, not a decompressive alternative;
• Attempting the procedure without sufficient microsurgical experience or inadequate surgical exposure.

**Surgical pearls**:

• Adequate microsurgical exposure of the basal cisterns is mandatory; incomplete opening limits physiological benefit [29,36,37];
• Irrigation should be performed gently to avoid secondary injury while facilitating clearance of subarachnoid blood;
• The goal is not drainage alone, but restoration of CSF circulation and perivascular flow;
• Failure to observe physiological improvement should prompt treatment escalation.

### 7.4. External Ventricular Drain

**Goal**:

To restore intracranial buffering capacity through controlled CSF diversion, thereby improving intracranial compliance and stabilizing the pressure–volume relationship. Ventriculostomy aims to unload the ventricular compartment, modulate CSF dynamics, and preserve cerebral perfusion when compliance failure is present but potentially reversible.

**Situations where it works best**:

• ICCS Stage 2, when intracranial compliance is impaired but cerebral oxygenation remains preserved;
• Patients in whom CSF diversion is expected to contribute meaningfully to intracranial hypertension control, such as those with post-traumatic hydrocephalus, intraventricular hemorrhage, or ventricular contribution to compliance failure;
• As an early therapeutic intervention for intracranial hypertension when indicated, provided it does not delay urgent decompressive or lesion-directed surgery.

**Pitfalls**:

• Using ventriculostomy as a definitive strategy in ICCS Stage 3, where compliance failure is associated with cerebral hypoxia and requires aggressive decompression;
• Relying on absolute ICP reduction without improvement in waveform morphology or global physiological response;
• Excessive or uncontrolled CSF drainage leads to ventricular collapse, hemorrhage, or misleading transient improvement;
• Delayed escalation when compliance continues to deteriorate despite adequate ventricular drainage.

**Figure 9:**
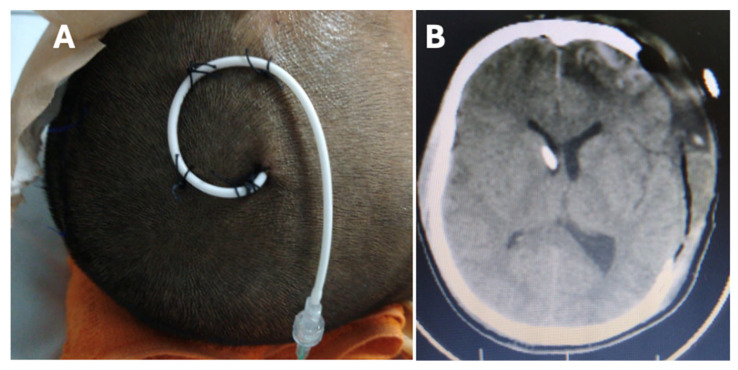
Panel (**A**) shows the external ventricular drain (EVD) tunneled subcutaneously and secured to the scalp with sutures. The catheter exit site is clean, and the system is connected to a closed sterile drainage circuit for intracranial pressure monitoring and cerebrospinal fluid diversion. Panel (**B**) shows a CT scan demonstrating the trajectory of the EVD inserted through a frontal approach (Kocher’s point). The catheter follows an appropriate intraparenchymal course, and the tip is correctly positioned within the frontal horn of the lateral ventricle, confirming adequate placement.

**Surgical pearls**:

• Prefer Kocher’s point; adjust only when anatomy or prior surgery mandates deviation;
• Depth is physiology-guided, not metric-guided: CSF return defines ventricular entry;
• Secure long subcutaneous tunneling (≥10 cm) to reduce infection and accidental displacement (see Figure 9);
• Initiate gradual CSF drainage; avoid rapid decompression that may precipitate ventricular collapse or hemorrhage;
• Always correlate drainage efficacy with ICP waveform pulsatility, not ICP values alone;
• Absence of drainage or waveform oscillation should trigger immediate patency assessment before imaging or escalation;
• When precise and continuous ICP assessment is essential, ventricular drainage should be complemented by an intraparenchymal ICP sensor, as ventricular drainage transducers alone may be unreliable.

## 8. Future Directions in Surgical Management of TBI

The ICCS-based approach should be regarded as a conceptual surgical framework rather than a validated protocol. Its purpose is to reorganize operative reasoning around intracranial compliance as a determinant of surgical timing and technique, which requires prospective validation. The operative rules in Section 5 and thresholds in Table 1 are intended for hypothesis generation and structured clinical reasoning, not as evidence-based protocols. No sensitivity, specificity, or prospective validation data exist for the staging combinations presented. Clinicians should integrate ICCS principles as a structured lens alongside—not instead of—established guideline-based care. Its relationship to DECRA and RESCUEicp is complementary: the ICCS framework does not contradict those findings but proposes a physiological substrate through which outcome variability in surgical trials can be better understood and through which future trials can design more targeted enrollment criteria [20,21].

Future developments should prioritize direct or real-time assessment of intracranial compliance, including emerging non-invasive technologies such as Brain4Care, which allow ICP waveform analysis and early detection of compliance failure before overt perfusion collapse occurs. In parallel, artificial intelligence models integrating imaging, neuromonitoring, and physiological data may assist in predicting the need for surgical escalation.

Finally, incorporation of ICCS staging into clinical trial design may improve patient stratification and enable surgical strategies to be tested based on physiological phenotype rather than uniform numerical thresholds. Future trials should consider ICCS-based stratification as a pre-specified enrollment criterion, selecting patients by compliance stage rather than uniform ICP or lesion-volume thresholds. Primary endpoints must extend beyond mortality to include validated functional recovery instruments such as the Extended Glasgow Outcome Scale (GOSE) and Disability Rating Scale (DRS) at 6 and 12 months. This is a recognized limitation of the current conceptual framework: the ICCS staging system requires prospective clinical validation with functional outcome tracking across multiple centers before it can be adopted as a clinical protocol. Multicenter registries that systematically collect compliance indicators alongside surgical decisions and functional outcomes would provide the foundational dataset needed to validate ICCS-based thresholds and escalation triggers. Recent work exploring compliance-centered approaches to neurosurgical decision-making provides additional theoretical grounding for this translational endeavor [38,39].

## 9. Conclusions

Historically, surgical decision-making in TBI has relied on fixed volumetric criteria and intracranial pressure thresholds. However, what ultimately drives neurosurgical intervention is not numerical values, but the loss of intracranial compliance and the physiological consequences that follow. Importantly, this failure of compliance can and should be recognized before overt neurological deterioration becomes clinically apparent.

The ICCS-based framework does not replace clinical judgment; it structures it by shifting surgical reasoning from lesion-centered thresholds to physiology-centered assessment.

Likewise, this compliance-based model does not challenge randomized evidence but offers physiological lens through which existing surgical outcome data can be interpreted more coherently, helping explain outcome variability, refine patient selection, and guide future trial design within a more accurate biological framework.

## Figures and Tables

**Table 1:** Characteristic findings are associated with each stage of ICCS. ONSD = Optic Nerve Sheath Diameter; NPi = Neurological Pupil Index; QPi = Quantitative Pupillary Index; MCA = Middle Cerebral Artery; PI = Pulsatility Index; FVd = End-Diastolic Velocity; NIRS = Near Infrared Spectroscopy; PbtO_2_ = Partial Brain Tissue Oxygen Pressure.

Stage	Characteristics
Stage 1	Abnormal CT scan (brain edema or intracranial hemorrhage) Normal ICP waveform Normal brain oxygenation (NIRS > 50% or PbtO_2_ > 20 mmHg) NPi/QPi > 3 ONSD < 6 mm MCA TCD PI < 1.3
Stage 2	Abnormal CT scan (brain edema or intracranial hemorrhage) Abnormal ICP waveform (P2 > P1) Normal brain oxygenation (NIRS > 50% or PbtO_2_ > 20 mmHg) NPi/QPi < 3 ONSD > 6 mm MCA TCD PI > 1.3 with FVd < 20 cm/sec
Stage 3	Abnormal CT scan (brain edema or intracranial hemorrhage) Abnormal ICP waveform (P2 > P1) Abnormal brain oxygenation (NIRS < 50% or PbtO_2_ < 20 mmHg) NPi/QPi < 3 ONSD > 6 mm MCA TCD PI > 1.3 with FVd < 20 cm/sec

## Data Availability

No new data were created or analyzed in this study.
